# Decrease in Proportion of CD19^+^CD24^hi^CD27^+^ B Cells and Impairment of Their Suppressive Function in Graves’ Disease

**DOI:** 10.1371/journal.pone.0049835

**Published:** 2012-11-26

**Authors:** Bingbing Zha, Luman Wang, Xiaoming Liu, Jun Liu, Zaoping Chen, Jiong Xu, Li Sheng, Yiming Li, Yiwei Chu

**Affiliations:** 1 Department of Immunology and Biotherapy Research Center, Shanghai Medical College, Fudan University, Shanghai, China; 2 Department of Endocrinology and Metabolism, Shanghai Fifth People’s Hospital, Fudan University, Shanghai, China; 3 Department of Endocrinology and Metabolism, Huashan Hospital, Fudan University, Shanghai, China; University of Southern California, United States of America

## Abstract

IL-10-producing B cells (B10 cells) have been shown to play a suppressive role in the peripheral blood of humans, with their numbers and function altered in several autoimmune diseases. However, the role of B10 cells in Graves’ disease (GD) remains unknown. In this study, we demonstrated that B10 cells in human peripheral blood belonged to a CD24^hi^CD27^+^ B cell subpopulation. The proportion of B10 cells along with the CD19^+^CD24^hi^CD27^+^ B cell subset was significantly lower in new-onset patients compared with healthy individuals. In recovered patients, these proportions were restored to levels similar to those seen in healthy individuals. Additionally, we found that CD19^+^CD24^hi^CD27^+^ B cells from healthy individuals inhibited proliferation and TNF-α production of CD4^+^ T cells via an IL-10–independent pathway. They also inhibited IFN-γ production by CD4^+^ T cells, through an IL-10–dependent pathway. In contrast, their suppressive function on CD4^+^ T cell proliferation and cytokine production was impaired in new-onset and recovered patients compared with healthy individuals. Our study provides evidence that CD19^+^CD24^hi^CD27^+^ B cells may possess the capacity to downregulate immune responses, partially by production of IL-10 in human peripheral blood. Impairment of their immunosuppressive function may contribute to GD pathogenesis and relapse.

## Introduction

Graves’ disease (GD) is an organ-specific autoimmune disorder characterized by the loss of immunological tolerance, which is pivotal to the appearance of pathogenic autoantibodies against thyroid peroxidase (TPO), thyroglobulin (Tg), and the TSH receptor (TSHR) [1], [2]. This leads to secretion of thyroid hormone with resulting hyperthyroidism and goiters [3]. In some GD patients, intense reactive T and B lymphocytes infiltrate the thyroid and are not effectively suppressed by peripheral tolerance mechanisms [4]. However, the exact etiology of this disease is not well understood, with the consequence that treatment of Graves’ disease has not changed over the past 50 years [5]. Immunotherapy until now has included polarization of the Th1/Th2 balance of T-helper cells, targeting regulatory T cells and influencing APC-T cell interactions [6]. However these therapies have only been applied to modify the course of GD in animal models. Recently, clinical trials of a B-lymphocyte depleting monoclonal antibody, anti-CD20 rituximab (RTX), for GD patients have been initiated. B lymphocytes comprise around 9% of lymphocytes in the thyroid gland and in the peripheral blood of GD patients. RTX depletes circulating B lymphocytes efficiently, but the effect is moderate, with only 40% of patients recovering [7], [8], [9]. Moreover, Goetz found disease was exacerbated when RTX was used in patients with ulcerative colitis [10], and Klaus Lehmann-Horn found out that B cell depletion accentuated pro-inflammatory reaction in neuroimmunological disorders [11], these findings suggesting that B cells could also have regulatory roles in human blood. We hypothesized that B cell depletion therapy in GD patients eliminated regulatory B cells in peripheral blood, simultaneously resulting in a lower recovery rate. We also postulated that regulatory B cells might be associated with the etiology of GD.

IL-10-producing regulatory B (B10) cells have been identified to play a protective role in murine models of autoimmune diseases, including collagen-induced arthritis (CIA) [12],experimental autoimmune encephalomyelitis (EAE) [13], diabetes [14], contact hypersensitivity (CHS) [15], allergic airway inflammation [16], and intestinal mucosal inflammation [17]. The phenotype and molecular mechanisms of B10 cells in mice have been comprehensively studied. In a murine model of autoimmune disease, the regulatory function of B10 cells observed in *ex vivo* suppressive assays and in *in vivo* adoptive transfers was partially IL-10 dependent [18], [19], [20]. Recently, several studies have provided evidence that human B cells can also regulate inflammatory responses. Blair and colleagues found that CD24^hi^CD38^hi^ immature B cell population contained B10 cells that inhibited TNF-α production by CD4^+^ T cells, and blockade of IL-10 and IL-10R completely reversed their suppressive ability. They also found that CD24^hi^CD38^hi^ B cells were functionally impaired in Systemic Lupus Erythematosus (SLE) patients [21]. In contrast, Iwata et al demonstrated that IL-10-producing B cells were identified as CD24^hi^CD27^+^ B cells. The frequency of these cells was increased in most autoimmune disease patients. Human B cells can inhibit the function of CD4^+^ T cells *via* IL-10-independent pathways [22]. Therefore, the phenotype and immune regulatory mechanisms of IL-10-producing human B cells remain controversial, with more research required to elucidate their phenotype and regulatory mechanisms.

In this study we aimed at identifying the frequency, phenotype and function of B10 cells in healthy individuals and GD patients. We found that *ex vivo* B10 cells predominantly belonged to the CD24^hi^CD27^+^ B cell subpopulation in GD patients and healthy individuals. The frequency of B10 cells and the CD19^+^CD24^hi^CD27^+^ subset was significantly lower in new-onset GD patients compared with that in healthy individuals, while frequencies were similar in healthy individuals and recovered patients. Furthermore, we demonstrated a suppressive function of CD19^+^CD24^hi^CD27^+^ cells on CD4^+^ T cell proliferation, with cytokine production impaired in GD patients.

## Results

### Enumeration of IL-10-producing B cells in Healthy Individuals and GD Patients

**Figure 1:**
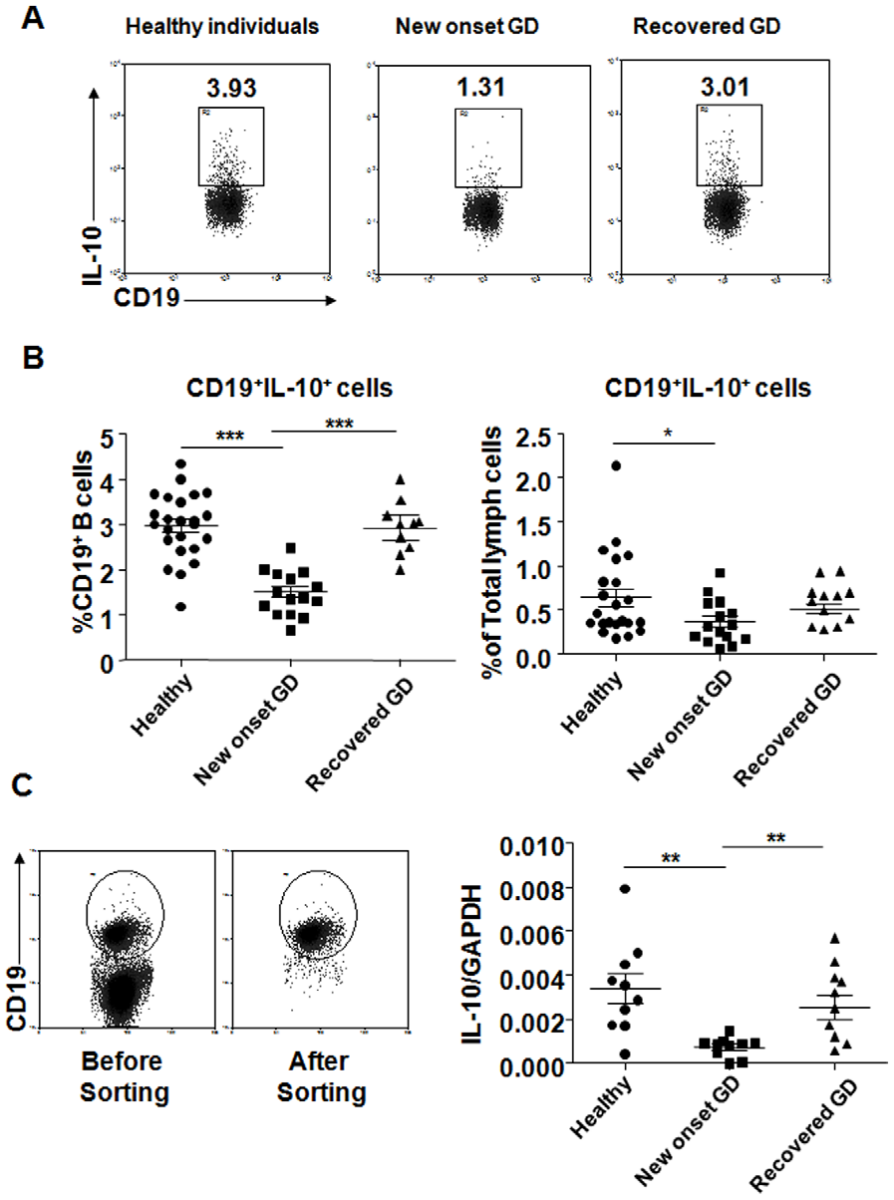
Frequencies of B10 cells from the blood of healthy individuals and GD patients. (A) Representative intracellular IL-10 expression in CD19^+^ B cells of healthy individuals, new-onset GD patients, and recovered GD patients after *in vitro* CpG+PIB stimulation for 5 h. (B). Dots represent B10 cell frequencies in CD19^+^ B cells and total PBMCs after *in vitro* CpG+PIB stimulation from 24 healthy individuals, 15 new-onset GD patients, and 10 recovered GD patients. (C) IL-10 mRNA transcript expression in CD19^+^ B cells. The RNA of freshly purified CD19^+^ B cells was isolated (left) and IL-10 transcripts quantified by quantitative real-time PCR. Dots represent results from 10 individuals of each group (right). *P<0.05, **P<0.01 and ***p<0.0001. Columns and error bars represent mean±SEM.

Human blood B10 cells were defined as B cells expressing cytoplasmic IL-10 after 5 h of stimulation with CpG, PMA, ionomycin and BFA. We counted the frequency of B10 cells in total PBMCs and B cells respectively. In new-onset GD patients (*n* = 15), B10 cells compose 0.36% of total PBMCs and 1.51% of B cells, in recovered GD patients (n = 10), 0.51% of total PBMCs and 2.92% of B cells, and, in healthy individuals (*n* = 24), 0.64% of total PBMCs and 2.97% of B cells, respectively. (Fig. 1A, B). The frequency of B10 cells was significantly lower in new-onset GD patients compared with healthy individuals. This frequency in recovered GD patients was increased and close to the level observed in healthy individuals. We also assessed IL-10 transcripts quantification in blood CD19^+^ B cells. In new-onset GD patients, the levels of these transcripts were 2–3 folds lower than those in healthy individuals and in recovered GD patients. The levels of IL-10 transcripts were similar in recovered patients and healthy individuals (Fig. 1C).

### Phenotypic Characterization of IL-10-competent B cells and Enumeration

**Figure 2:**
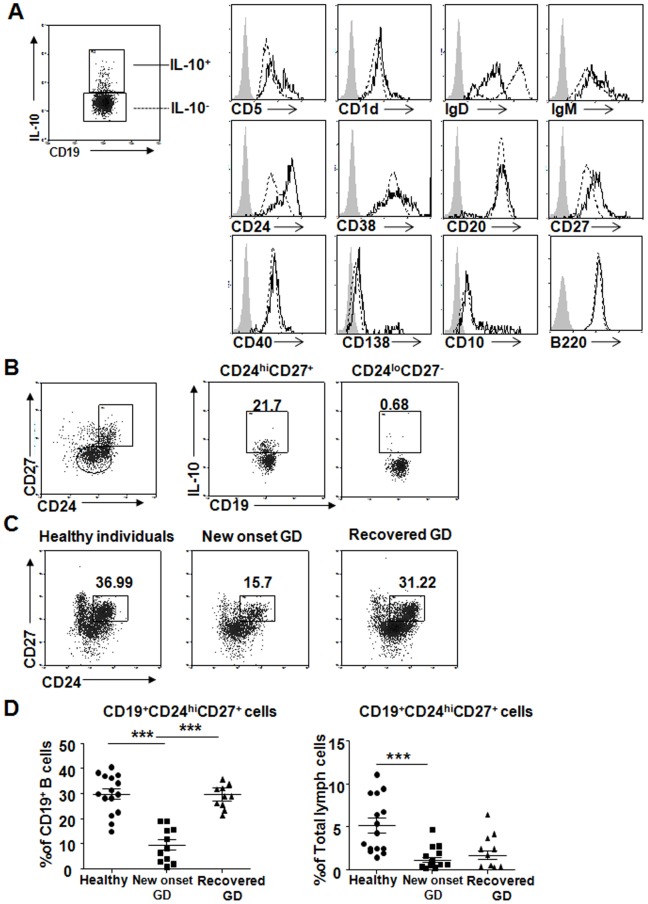
Phenotype of B10 cells and enumeration of this subpopulation in healthy individuals and GD patients. (A) Representative cell surface phenotypic analysis of IL-10+ (solid line) or IL-10− (dashed line) B cells was picked from healthy individuals. (B) The CD24^hi^CD27^+^ subpopulation was the predominant B10 subset. B cells purified from the blood of healthy individuals were cultured with CpG+PIB for 5 h before permeabilization and staining using CD19, CD24, and CD27 cell-surface monoclonal antibodies and a cytoplasmic IL-10 monoclonal antibody. This was followed by flow cytometry analysis. B10 cells within the CD24^hi^CD27^+^ and CD24^lo^CD27^−^ subpopulations is shown, with the proportions of IL-10 cells within the indicated gates shown. (C) CD19^+^CD24^hi^CD27^+^ frequencies from fresh blood cells of healthy individuals, new-onset GD patients and recovered GD patients. (D) The CD19^+^CD24^hi^CD27^+^ cell subpopulation in individuals as in (C). Dots represent CD19^+^CD24^hi^CD27^+^ frequencies in B cells and in total PBMCs from 15 healthy individuals, 11 new-onset GD patients, and 10 recovered GD patients. ***p<0.0001. Columns and error bars represent mean±SEM.

As described before, the phenotype of human B10 cells remains ill-defined. Their phenotypic characterization was determined by analyzing cell surface markers (IgM, IgD, CD1d, CD5, CD10, CD20, CD24, CD27, CD38, CD40, CD138 and B220) after in vitro stimulation with CpG and PIB for 5 h. As shown in Fig. 2A and Fig. S1, blood B10 cells expressed low IgD levels and high CD24 and CD27 levels. The remaining cell surface markers were expressed at similar levels on both IL-10^+^ B cells and IL-10^−^ B cells. As shown in Fig. 2B, blood B10 cells were predominantly found within the CD24^hi^CD27^+^ B cell subset in both healthy individuals and GD patients. There is no much difference in the phenotype of human blood B10 cells between healthy control and GD patients. Representative cell surface phenotypic analysis of IL-10^+^ or IL-10^−^ B cells was picked from one of the healthy individuals in figure 2A and 2B. The percentage of CD19^+^IL-10^+^ B cells in the CD24^hi^CD27^+^ subset was 21.7%, but 0.68% in the CD24^lo^CD27^−^ subset. Furthermore, we counted the frequency of CD24^hi^CD27^+^ subset in total PBMCs and B cells respectively. In new-onset GD patients (*n* = 11), CD19^+^CD24^hi^CD27^+^ subset composed 1.09% of total PBMCs and 9.68% of B cells, in recovered GD patients (*n* = 10), 1.64% of total PBMCs and 29.95% of B cells, and in healthy individuals (*n* = 15), 5.13% of total PBMCs and 30.09% of B cells, respectively. (Fig. 2C, D).The frequency of the CD19^+^CD24^hi^CD27^+^ subset was significantly lower in new-onset GD patients compared with healthy individuals; however, frequencies were increased for recovered GD patients.

### CD19^+^CD24^hi^CD27^+^ B cells Regulate CD4^+^ T cell Proliferation and Cytokine Production

**Figure 3:**
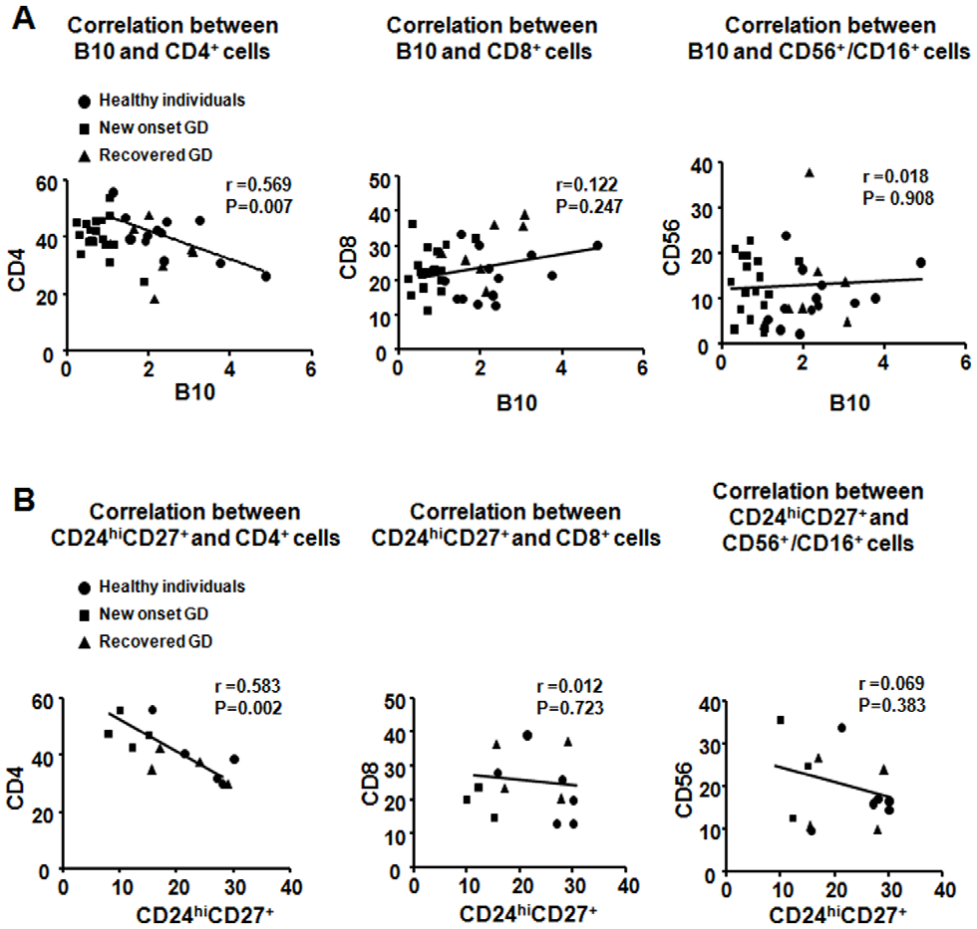
CD4^+^ cell frequency is negative related to B10 cell frequency. (A) Relative frequencies of CD4^+^ cells, CD8^+^ cells, CD16^+^CD56^+^ cells and B10 cells in healthy individuals and GD patients. (B) Relative frequencies of CD4^+^ cells, CD8^+^ cells, CD16^+^CD56^+^, and CD19^+^CD24^hi^CD27^+^ cells identified in healthy individuals and GD patients.

To further demonstrate the target cell of regulatory B cells, a link between the frequencies of regulatory B cells and the frequencies of other effect lymph cells, including CD4^+^ T cells, CD8^+^ T cells, and CD16^+^CD56^+^ NK cells, has been found in form of a negative relationship. We found that a significant negatively correlated between B10 cell frequencies and CD4^+^ cell frequencies (*n* = 36, *P*<0.001), however, no correlation between B10 cell and other lymph cell such as CD8^+^ T cell, CD16^+^CD56^+^ NK cells (Fig. 3A). And also, there was a significant negative relationship between CD19^+^CD24^hi^CD27^+^ cell frequencies and CD4^+^ cell frequencies (*n* = 13, *P* = 0.0024) (Fig. 3B), and no correlation between CD19^+^CD24^hi^CD27^+^ cell and CD8^+^ T cell or CD16^+^CD56^+^ NK cells. Significant negative correlation may render CD4^+^ cells more sensitive to regulatory B cells than other effect lymph cells. Thus, we isolated B10 cells with the help of CD19, CD24 and CD27 markers, and analyzed whether CD19^+^CD24^hi^CD27^+^ B cells could regulate CD4^+^ T cell proliferation and cytokine production.

**Figure 4:**
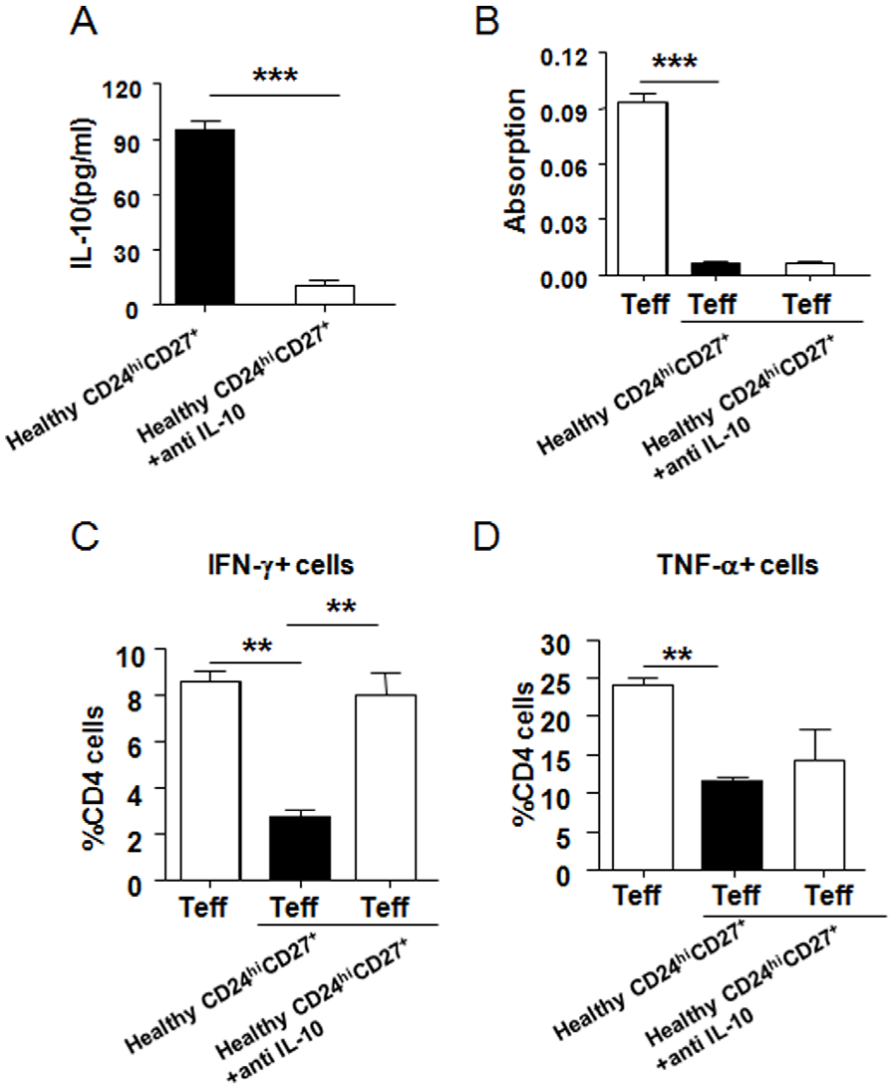
CD19^+^CD24^hi^CD27^+^ B cells from healthy individuals regulate CD4^+^ T cell proliferation and cytokine production. (A) Purified CD19^+^CD24^hi^CD27^+^(CD24^hi^CD27^+^) B cells isolated from blood were cultured with CD40L+CpG, in the presence or absence of blocking antibodies to IL-10. Any IL-10 secreted into the culture supernatant was quantified by ELISA. (B) Purified CD4^+^ cells from the blood of healthy individuals were cultured with media alone or purified CD19^+^CD24^hi^CD27^+^ B cells, then stimulated with 0.5 mg/ml of a plate-bound CD3 monoclonal antibody and CD40L+CpG with or without blocking antibodies to IL-10. Proliferation was assessed using Brdu 120 h later. After 120 h, the CD4^+^ cells as in (B) were stained for IFN-γ and TNF-α. Expression of TNF-α and IFN-γ was assessed by flow cytometry. The bar chart shows cumulative data as mean±SEM of three or more independent experiments. Compared to healthy CD24^hi^CD27^+^ group, **P<0.01, ***p<0.0001.

**Figure 5:**
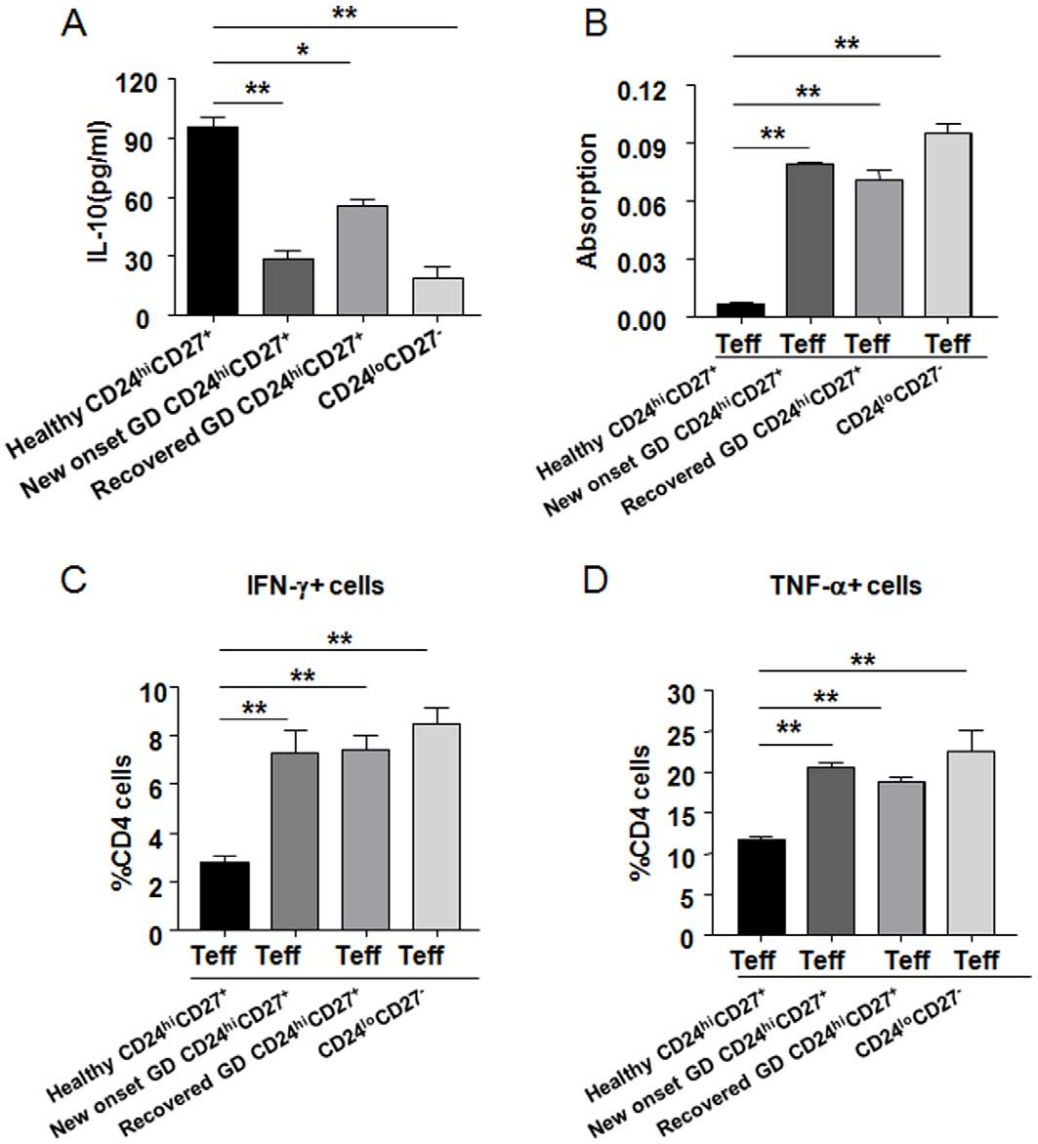
CD19^+^CD24^hi^CD27^+^ B cells from GD patients fail to suppress CD4^+^ cell proliferation and cytokine secretion. (A) Purified CD19^+^CD24^hi^CD27^+^ B cells (CD24^hi^CD27^+^) from healthy individuals, new-onset and recovered GD patients or CD19^+^CD24^lo^CD27^−^ B cells (CD24^lo^CD27^−^) from healthy individuals cultured with CD40L+CpG. CD19^+^CD24^hi^CD27^+^ B cells from new-onset and recovered GD patients produced less IL-10. The suppressive effect of CD19^+^CD24^hi^CD27^+^ B cells on CD4^+^ T cell proliferation (B) and cytokine secretion (C,D) were significantly impaired in both new-onset and recovered GD patients compared with healthy individuals. The bar chart shows cumulative data as mean±SEM of three or more independent experiments. Compared to healthy CD24^hi^CD27^+^ group, *P<0.05 and **P<0.01.

CD19^+^CD24^hi^CD27^+^ cells of over 95% purity from the blood of healthy individuals were cultured with isolated CD4^+^ T cells from healthy individuals in the presence of CpG, CD40L and a plate-bound CD3 monoclonal antibody, with or without an IL-10 monoclonal antibody (data not shown). B10 cells represented 21.7% of the blood CD24^hi^CD27^+^ B cell subset, while B10 cells represented less than 1% of non-CD24^hi^CD27^+^ B cells. Therefore, 2×10^5^ CD24^hi^CD27^+^ B cells included approximately 4.34×10^4^ IL-10-competent B10 cells. After 5 days, the proliferation of CD4^+^ T cells was assessed. In healthy individuals, CD19^+^CD24^hi^CD27^+^ B cells produced a large amount of IL-10 (Fig. 4A, 5A), inhibiting the proliferation of CD4^+^ T cells. The addition of an anti-IL-10 monoclonal antibody did not reverse the inhibition of CD4^+^ T cells proliferation (Fig. 4B). The ability of CD19^+^CD24^hi^CD27^+^ B cells to inhibit pro-inflammatory cytokine production in CD4^+^ T cells was also assessed by flow cytometry. CD4^+^ T cells co-cultured with CD19^+^CD24^hi^CD27^+^ B cells resulted in significant reduction of IFN-γ and TNF-α production (Fig. 4C, D). IFN-γ production was remarkably blocked by anti–IL-10 monoclonal antibody, whereas TNF-α production was not reversed by anti–IL-10 monoclonal antibody in healthy individuals (Fig. 4C, D).

### CD19^+^CD24^hi^CD27^+^ B cells from GD Patients Fail to Suppress CD4^+^ cell Proliferation and Cytokine Secretion

Our studies have revealed that the proportion of CD19^+^CD24^hi^CD27^+^ B cells was significantly decreased in new-onset GD patients. This proportion was increased in cured patients, to a level similar to that observed in healthy individuals. We explored the possibility that, in GD disease, this subset of B cells may regulate the proliferation and cytokine production in CD4^+^ T cells. Interestingly, CD19^+^CD24^hi^CD27^+^ B cells from new-onset and recovered GD patients produced less IL-10 compared with healthy individuals (Fig. 5A). The suppressive effect of CD19^+^CD24^hi^CD27^+^ B cells on CD4^+^ T cell proliferation was significantly impaired in both new-onset and recovered GD patients compared with that in healthy individuals (Fig. 5B). Additionally, in new-onset and recovered GD patients, the ability of CD19^+^CD24^hi^CD27^+^ B cells to inhibit the production of IFN-γ and TNF-α in CD4^+^ T cells was also significantly impaired (Fig. 5C, D).The suppressive function of CD19^+^CD24^hi^CD27^+^ B cells in new-onset and recovered GD patients were very similar to CD19^+^CD24^lo^CD27^−^ B cells.

## Discussion

Graves’ disease (GD) is an autoimmune disease with deficiencies of immunological tolerance. The frequency and role of regulatory T cells (Tregs) in GD patients remains controversial. Marazuela et al showed that Tregs are deficient and functionally impaired in autoimmune thyroid diseases [4]; However, in 2009 Pan found that Tregs were not deficient in the peripheral blood of active GD patients and GD patients post-treatment [23]. Thus, further study was needed to determine the reason for the abnormal immune tolerance in GD. It has emerged that a specific IL-10-producing subset of B cells downregulate immune responses. We quantified the number of B10 cells, by exploiting their ability to express cytoplasmic IL-10 after 5 h of *ex vivo* stimulation. Stimuli included PMA, ionomycin, and CpG, which induced cytoplasmic IL-10 expression and secretion in human B10 cells. This was then able to be easily quantified *in vitro* [22]. Our combined studies describe that B10 cells were predominantly found within the CD24^hi^CD27^+^ B cell subpopulation. There was a numerical and functional reduction of CD19^+^CD24^hi^CD27^+^ B cells in patients with GD. They inhibited proliferation and TNF-α production of CD4^+^ T cells through IL-10-independent pathways, and inhibited IFN-γ through IL-10-dependent pathways *in vitro.* Therefore, CD19^+^CD24^hi^CD27^+^ B cells regulate immune responses partially by secretion of IL-10, and probably via other ways, for instance generate Ag-specific Abs or induced increase in Tregs as describe before [24], [25].

Our work demonstrated that the frequency of B10 cells, and IL-10 mRNA expression in B cells were significantly lower in new-onset GD patients compared with healthy individuals. The frequencies were increased in recovered patients, to a level similar to that seen in healthy individuals. In contrast Iwata et?al found that the mean B10 cell frequencies in blood were significantly higher in patients with autoimmune diseases such as rheumatoid arthritis, systemic lupus erythematosus, autoimmune vesiculobullous skin disease, or multiple sclerosis when compared with healthy individuals [22]. The patients recruited in those studies had been undergoing active treatment with immunomodulatory agents and/or low doses of prednisone, possibly influencing the frequency of B10 cells. Our research has taken into consideration the likely influence of drugs and clinical status, with all new-onset and recovered patients not receiving any anti-thyroid drug treatment. Recovered patients were in a euthyroid state for at least 12 months. Patient cohorts were needed to fully assess the relationship of B10 cell numbers with clinical and laboratory data. We observed that B10 cell frequencies negatively correlated with CD4^+^ lymphocyte frequencies in the peripheral blood of GD patients. This would imply that effecting of CD4^+^ T lymphocytes are not effectively suppressed by the peripheral tolerance mechanisms of B10 cells, which can result in hyper-proliferation of CD4^+^ T lymphocytes in GD patients.

IL-10–competence remains the best phenotypic marker for defining human B10 cells. The objective of our study was to determine whether human B10 cells belonged to a distinct subpopulation, or appeared from any B cell population following appropriate activation stimuli. Two populations of B10 cells with suppressive capacities have been shown to exist in human peripheral blood. Blair et al demonstrated that human CD19^+^CD24^hi^CD38^hi^ B cells possessed regulatory capacity [21], while Iwata et al showed that B10 cells were predominantly found within the CD24^hi^CD27^+^ B cell subpopulation [22]. Bouaziz et al postulated that B10 cells did not fall within a single clearly defined subpopulation, but were enriched in both the memory (CD27^high^) and transitional (CD38^high^) B-cell compartments [26]. Based on the observations made in this study, we found that freshly isolated IL-10^+^ B10 cells from peripheral blood were predominantly found within the CD24^hi^ and CD27^+^ B-cell subpopulations. The frequencies of B10 cells were more than 30-fold higher within the CD24^ hi^CD27^+^ subpopulation compared with the CD24^lo^CD27^−^ B cell subpopulation. CD27 is a widely used marker for memory B cells and CD24 is expressed on almost all B cells [27], [28]. CD24^hi^CD27^+^ and CD24^lo^CD27^+^ cells represent memory B cells and mature B cells, respectively [29], [30]. Thus, CD24^lo^CD27^+^ cells may have the potential to differentiate into CD24^hi^CD27^+^ cells. We and other groups determined that human IL-10 producing B cells in the blood were predominantly CD24^hi^CD27^+^, while CD24^lo^CD27^−^ B cells that represent immature or naïve B cells were not IL-10 producing cells [22]. CD24^lo^CD27^+^ cells that obviously exist in our flow data were not IL-10 producing cells either, but maybe have contain progenitor IL-10 producing cells, thus a deficiency in CD24^lo^CD27^+^ cells may explain the lack of progenitor IL-10 producing cells, which also deficient in new onset GD patients’ blood (data not shown). Many B10 cells were also found in the CD38^+^ B cell subpopulation. B10 cell frequencies were more than 20-fold higher within the CD24^hi^CD38^hi^ subpopulation compared with the CD24^lo^CD38^lo^ B cell subpopulation (data not shown). Therefore, we believe that CD24^hi^CD27^+^ is a slightly better marker than CD24^hi^CD38^hi^ and could be used to effectively isolate a subset of B10 cells from human peripheral blood.

We next evaluated whether there was a deficiency in the number of CD19^+^CD24^hi^CD27^+^ B cells in GD patients. We observed significantly lower percentages of CD19^+^CD24^hi^CD27^+^ B cells in new-onset GD patients compared with healthy individuals. We also found that although the frequency of CD24^hi^CD27^+^ cells in CD19^+^ B cells from recovered patients were restored to normal levels seen in healthy individuals, but, their proportion in total PBMCs were obviously lower than in healthy controls. Our previous studies showed that the frequency of CD19^+^ B cells was increased in new onset patients compared to healthy controls, and that the expanded cells were mostly CD19^+^IL-10^–^ cells, while in recovered patients, this proportion was decreased to levels similar to healthy individuals. Thus, a relative deficit of total B cells in the recovered patients resulted in the decreasing proportions of CD19^+^CD24^hi^CD27^+^ B cells in total PBMCs. This phenomenon may be important in the pathogenesis of Graves’ disease (GD), but no evidence show that it can be an explanation for the IL-10 independent way. We also assessed the suppressive capacity of purified CD19^+^CD24^hi^CD27^+^ B cells on regulated proliferation and cytokine production of CD4^+^ T cells in healthy individuals. We show that CD19^+^CD24^hi^CD27^+^ B cells regulate proliferation of CD4^+^ T cells through IL-10-independent pathways. Bouaziz et al have also described that purified B cells activated with CpG and anti-immunoglobulins inhibited T-cell proliferation, and this was not reversed following IL-10 blocking. Furthermore, we also found that CD19^+^CD24^hi^CD27^+^ B cells regulates IFN-γ production of CD4+T cells through IL-10–dependent pathways and TNF-α production through IL-10 independent pathways. These results were similar to those described by Blair et al, who also stated that the CD24^hi^CD27^+^ B cell subpopulation was able to negatively regulate monocyte cytokine production through IL-10-dependent pathways [22]. IL-10 is known as human cytokine synthesis inhibitory factor (CSIF), and exhibits a very potent anti-inflammatory capacity. Recently, the mechanism of B cells inhibitory capacity has focused on their ability to produce IL-10, and have convincingly demonstrated that B cells producing IL-10 can limit disease in animal models of experimental autoimmune encephalomyelitis, inflammatory bowel disease, and rheumatoid arthritis. However, it has also been reported that B cells can inhibit disease in an IL-10-independent manner [31]. One possible IL-10-independent mechanism of B cell regulation of autoimmune diseases is via the production of antibodies. IgG is reported to bind to inhibitory FcRIIB which is widely expressed by dendritic cells (DCs) and suppresses immune responses through the activation of an ITIM [32], [33]. IgA and IgM produced by activated B cells and plasma cells in the walls of the gastrointestinal and respiratory tracts can be actively transported across epithelial cells into the lumens of these tracts, and perform anti-microbiota functions that can protect against colitis or lung injury [34]. However, as we mentiond before, Graves’ disease (GD) is an organ-specific autoimmune disorder characterized by the appearance of pathogenic autoantibodies against thyroid peroxidase (TPO), thyroglobulin (Tg), and the TSH receptor (TSHR). These autoantibodies-TPOAb, TGAb and TSHRAb are belonging to the immunoglobulin G(IgG) subclass [35]. Thus, antibodies were confirmed as having a pathogenic role in GD, so we suggest they probably do not have a protective role in this disease. The other possible mechanism is via the induction of regulatory T cells (Tregs) [36], [37], [38]. We detected CD4^+^CD25^+^CD127^−^Foxp3^+^ Treg cells in both healthy individuals and patients, and observed a trend for decreased Tregs in GD patients (Fig. S2). Since patient samples were limited, we could not perform in vitro coculture experiments. Although we have no explanation as to why Tregs in the patient samples were decreased, it may be related to the B cell suppressive role, but further experiments are required to confirm this point. From in vitro and adoptive transfer experiments, B cells have been shown to have suppressive properties by interacting with T cells and DCs and inducing the production of TGF-β or the promoting costimulatory molecule CD40/CD40L and CD28/B7 signaling [21], [39], [40], [41], [42]. However, we did not detect the expression of TGF-β or find any difference in B cell-induced suppressive in vitro experiments, when we used inhibitors of CD40 or B7 (data not shown), which is consistent with previous studies [36]. In summary, we think the most likely IL-10-independent mechanism is the proliferation and migration of Tregs induced by B cells.

Our reported findings demonstrate that the suppressive function of the CD24^hi^CD27^+^ B cell subpopulation on CD4^+^ T cell proliferation and cytokine production was impaired in new-onset and recovered GD patients. It has been previously found that B cell regulation of T cell proliferation is impaired in patients with SLE [21], [43], but unaffected in patients with RA and pSS [43]. Another notable finding was that although the frequencies of regulatory B cells from peripheral blood were normal from recovered GD patients,CD19^+^CD24^hi^CD27^+^ B cells from recovered patients produced less IL-10 and their suppressive capacity was impaired when compared with healthy individuals. Accordingly, a breakdown of regulatory B cell immunosuppressive functions on proliferation and pro-inflammatory cytokine production in CD4^+^ T cells may be responsible for GD pathogenesis. Incomplete recuperation from these immunosuppressive functions in recovered GD patient may lead to a GD relapse.

In conclusion, our current findings provide new data that define the phenotype and suppressive capacity of B10 cells from the peripheral blood of healthy individuals and GD patients. Presently, our data are insufficient to determine whether a deficiency in the numbers of B10 and CD19^+^CD24^hi^CD27^+^ cells is the cause of GD, as opposed to an effect of GD. It will be necessary to perform additional studies to fully elucidate the complex role of B10 cells in the pathogenesis and treatment process of GD. Increasing the quantity and improving the function of B10 cells might result in innovative B cell-based therapies for the treatment of GD.

## Materials and Methods

### Patients and Controls

Peripheral blood was obtained from 15 new-onset (mean, 39 years; range, 23–58 years) and 19 recovered GD patients (mean, 36 years; range, 22–52 years). Diagnosis was established by commonly accepted clinical and laboratory criteria for GD [44]. All new-onset GD patients had not received any anti-thyroid drug treatment, and all GD recovered patients were in a euthyroid state as a result of treatment with methimazole for at least 12 months. Exclusion criteria were: patients with a recent history of infection or evidence of tumor; patients on immunomodulatory drugs; and pregnancy within the last 12 months. Tables 1 and 2 summarize the characteristics of GD patients. Age-matched healthy individuals (*n* = 36; mean, 37 years; range, 21–54 years) were studied in parallel as controls. The medical ethics council of Shanghai Fifth People’s Hospital approved this study (Approval number 002, 2011); patients and healthy volunteers recruited in this study all provided informed consents.

**Table 1:** Summary of New-onset GD patients Clinical Data.

				Autoantibody		
Subject	Gender	Age(y)	Disease Remission (m)	TRAb	TPOAb	TGAb
1	F	48	3	21.93	39.40	40.60
2	F	52	3	5.36	66.30	44.60
3	F	23	0.3	3.82	158.00	73.60
4	F	48	0.5	18.41	600.00	32.00
5	M	39	2	6.63	766.00	43.30
6	F	27	6	37.89	150.00	474.70
7	F	35	6	17.94	1290.00	24.00
8	F	34	1	24.10	653.00	35.00
9	M	58	3	15.42	1200.00	29.70
10	M	44	1	2.92	1100.00	220.40
11	F	30	6	6.30	364.00	156.00
12	F	41	0.5	5.33	684.80	136.40
13	F	41	1	9.66	1300.00	500.00
14	F	25	1	5.79	1280.00	243.90
15	F	36	3	10.63	49.70	500.00

1. Abbreviations: TRAb, TSH receptor antibodies; TPOAb, anti-thyroid peroxidase antibodies; TGAb, anti-thyroglobulin antbodies; y, year; m, month.

2. Normal values: TRAb: 0–2 mIU/ml; TPOAb: 0–60 U/ml; TGAb: 0–60 U/ml.

**Table 2:** Summary of Recovered GD patients Clinical Data.

				Autoantibody		
Subject	Gender	Age(y)	Disease Remission (m)	TRAb	TPOAb	TGAb
1	M	52	12	9.30	453.80	169.10
2	M	42	56	8.71	1300.00	320.30
3	F	46	48	4.00	336.00	659.00
4	F	30	15	5.00	77.40	41.30
5	M	47	17	5.20	1200.00	45.30
6	F	33	96	8.50	785.00	69.00
7	F	41	120	6.30	1110.00	205.40
8	F	37	31	7.62	1260.00	500.00
9	F	25	20	7.40	97.10	499.60
10	M	40	74	6.00	547.00	358.00
11	M	37	20	5.38	1000.00	40.40
12	F	33	29	8.65	859.00	235.00
13	M	28	14	3.65	796.00	332.00
14	F	45	19	2.57	489.00	53.00
15	M	31	43	1.24	680.40	177.30
16	F	22	78	3.94	257.00	35.80
17	M	21	17	5.60	1220.00	500.00
18	F	32	12	4.21	945.00	413.00
19	F	22	13	1.96	1150.00	25.30

1. Abbreviations: TRAb, TSH receptor antibodies; TPOAb, anti-thyroid peroxidase antibodies; TGAb, anti-thyroglobulin antbodies; y, year; m, month.

2. Normal values: TRAb: 0–2 mIU/ml; TPOAb: 0–60 U/ml; TGAb: 0–60 U/ml.

### Cell Isolation

Peripheral blood mononuclear cells (PBMCs) were isolated using discontinuous Lymphoprep (Axis-Shield PoC As, Oslo, Norway) gradient centrifugation. Isolated PBMCs were resuspended in phosphate-buffered saline (PBS) containing 2% fetal bovine serum (FBS).

### Antibodies

Anti-human monoclonal antibodies included: CD8 (RPA-T8), CD16 (3G8), and CD56 (NCAM16.2) from BD Biosciences (San Diego, CA); CD4 (OKT4), CD210 (IL-10R,3F9), IL-10 (JES3-9D7), IL-10 (JES3-19F1), IFN-γ (4S.B3), TNF-α (MAb11), CD19 (HIB19), CD5 (UCHT2), CD24 (ML5), CD138 (ID4), CD1d (51.1), CD10 (HI10a), CD38 (HIT2), IgD (IA6-2), IgM (MHM-88), CD40 (5C3), CD20(2H7), CD27 (O323) and CD45 (B220,HI30) from Biolegend (San Diego, CA). Functional grade CD3 (OKT3), CD25 (BC96), CD127 (eBioRDR5), Foxp3(236A/E7), was obtained from eBioscience (San Diego, CA).

### Flow Cytometry

Cells obtained from healthy individuals, new-onset and recovered GD patients were incubated with normal mouse serum (eBioscience) before staining with a specific antibody. The following antibodies were used for surface staining: FITC-conjugated anti-CD4; PE-conjugated anti-CD8; PE-conjugated anti-CD16; FITC-conjugated anti-CD56; FITC-conjugated anti-CD24; PerCP/Cy5.5-conjugated anti-CD19; APC/cy7-conjugated anti-CD27; FITC-conjugated anti-CD4; PE-conjugated anti-CD127; APC-conjugated anti-CD25; Intracellular cytokine analysis by flow cytometry was conducted as previously described [22].

For IL-10 detection, isolated leukocytes or purified cells were resuspended (1×10^6^ cells/ml) with 10 µg/ml CpG ODN2006 (Invivogen), 50 ng/ml phorbol 12-myristate 13-acetate (PMA; Sigma), 1 µg/ml ionomycin (Sigma), and 1×brefeldin A (BFA; BioLegend) for 5 h. Fc receptors were blocked with normal mouse serum before cell surface staining. The following antibodies were used for surface staining: FITC-conjugated anti-CD5, -CD24 or -CD138; PE-conjugated anti-CD1d or -CD10; PerCP/Cy5.5-conjugated anti-CD19; PE/Cy7-conjugated anti-IgD or -CD38; APC-conjugated anti CD20; APC/Cy7-conjugated anti-CD27; and Pacific blue-conjugated anti-IgM or -CD40. Cells were fixed and permeabilized according to the manufacturer’s instructions. Permeabilized cells were stained with PE- or APC-conjugated IL-10 monoclonal antibodies. For Foxp3 detection, Pacific blue-conjugated anti-Fopx3 was used, and cells were fixed and permeabilized according to the manufacturer’s instructions. For IFN-γ and TNF-α detection, cells were resuspended (1×10^6^ cells/ml) with 50 ng/ml PMA, 500 ng/ml ionomycin, and 1×BFA for 6 h. The Fc receptors were blocked with normal mouse serum before cell surface staining. The FITC-conjugated anti-CD4 antibody was used for surface staining. Cell were fixed and permeabilized according to the manufacturer’s instructions. Permeabilized cells were stained with an APC-conjugated TNF-α monoclonal antibody or Pacific blue-conjugated IFN-γ.

### Il10 Transcript Expression

CD19^+^ B cells were isolated from PBMCs using a Moflo FACS sorter with purities of 95–98%. Total RNA was isolated from purified cells using spin columns (Applied Biosystems, Foster City, CA). Total RNA from each sample was reverse transcribed into cDNA. The expression of IL-10 mRNA was analyzed using quantitative real-time polymerase chain reaction (PCR) methods according to the manufacturer’s instructions (Applied Biosystems). The sense GAPDH primer was 5′-GCC ACC CAG AAG ACT GTG GAT GGC-3′ and the antisense primer was 5′-CAT GTA GGC CAT GAG GTC CAC CAC-3′. The sense IL-10 primer was 5′-CTT CGA GAT CTC CGA GAT GCC TTC-3′ and the antisense primer was 5′-ATT CTT CAC CTG CTC CAC GGC CTT-3′.

### Cell Sorting and in vitro Cell Functional Assays

Isolated PBMCs were incubated with normal mouse serum before staining with a specific antibody. We used FITC-CD24, PerCP/Cy5.5-CD19, and APC Cy7-CD27 or FITC-CD4 for surface staining. The CD19^+^CD24^hi^CD27^+^ B cells and CD4^+^ T cells were isolated using a Moflo FACS sorter (ADP Analyzer; Beckman Coulter) with purities of 90–95%. After purification, CD4^+^ T cells were co-cultured with CD19^+^CD24^hi^CD27^+^ B cells in the presence of 10 µg/ml CpG, 1 µg/ml CD40L (R&D) and 1 µg/ml anti-CD3. Cells were cultured in 96-well plates for 5 days in complete RPMI 1640 (Gibco) containing 10% FBS, 2 mM L-glutamine, 0.05 mM 2-mercaptoethanol, and 100 U/ml of penicillin and streptomycin. Ionomycin, PMA and BFA were added for the last 6 hours as described previously [21]. Cells were then harvested and CD4^+^IFN-γ^+^ or CD4^+^TNF-α^+^ cells were detected by flow cytometry. For T cell proliferation analysis, BrdU was added for the last 5 hours and detected according to the manufacturer’s instructions for the BrdU ELISA colorimetric assay (Roche).

### Statistical Analysis

All statistical analysis was performed by Prism 5.0. Results were expressed as mean±SEM. The unpaired two-tailed Student t test for two data sets, or one-way ANOVA for three or more data sets, and the Spearman correlation to evaluate the relationship with B10 cells, CD19^+^CD24^hi^CD27^+^ B cells and other immunocytes. *P* values<0.05 were considered statistically significant.

## Supporting Information

Figure S1 **Mean fluorescence intensities of the surface markers between IL-10+ and IL-10neg B cells.** The mean intensity of fluorescence (MIF) of CD1d, CD5, IgD, IgM, CD24, CD38, CD20, CD27, CD40, CD138, CD10 and B220 expression was analyzed by flow cytometry. Dots represent results of cell surface phenotypic analysis of IL-10^+^ (solid line) or IL-10^−^ (dashed line) B cells. Column and error bars represent mean±SEM. **p<0.001.
(DOC)

Figure S2 **Frequencies of CD4^+^CD25^hi^CD127^lo^Foxp3^+^ Tregs from the blood of healthy individuals and GD patients.** (A) Representative intracellular Foxp3 expression in CD4^+^CD25^hi^CD127^lo^ Tregs of healthy individuals, new-onset GD patients, and recovered GD patients. (B) Dots represent CD4^+^CD25^hi^CD127^lo^ Tregs frequencies in CD4^+^ T cells and total PBMCs from 5 healthy individuals, 5 new-onset GD patients, and 5 recovered GD patients, respectively. Column and error bars represent mean±SEM.
(DOC)
