# Supplementary material for: Decrease in Proportion of CD19+CD24hiCD27+ B Cells and Impairment of Their Suppressive Function in Graves’ Disease
Source: PLoS One. 2012 Nov 26;7(11):e49835. doi: 10.1371/journal.pone.0049835 (PMC3506658; doi:10.1371/journal.pone.0049835)
Supplement: Figure S1 — Mean fluorescence intensities of the surface markers between IL-10+ and IL-10neg B cells. The mean intensity of fluorescence (MIF) of CD1d, CD5, IgD, IgM, CD24, CD38, CD20, CD27, CD40, CD138, CD10 and B220 expression was analyzed by flow cytometry. Dots represent results of cell surface phenotypic analysis of IL-10+ (solid line) or IL-10− (dashed line) B cells. Column and error bars represent mean±SEM. **p<0.001. (DOC) [file pone.0049835.s001.doc]

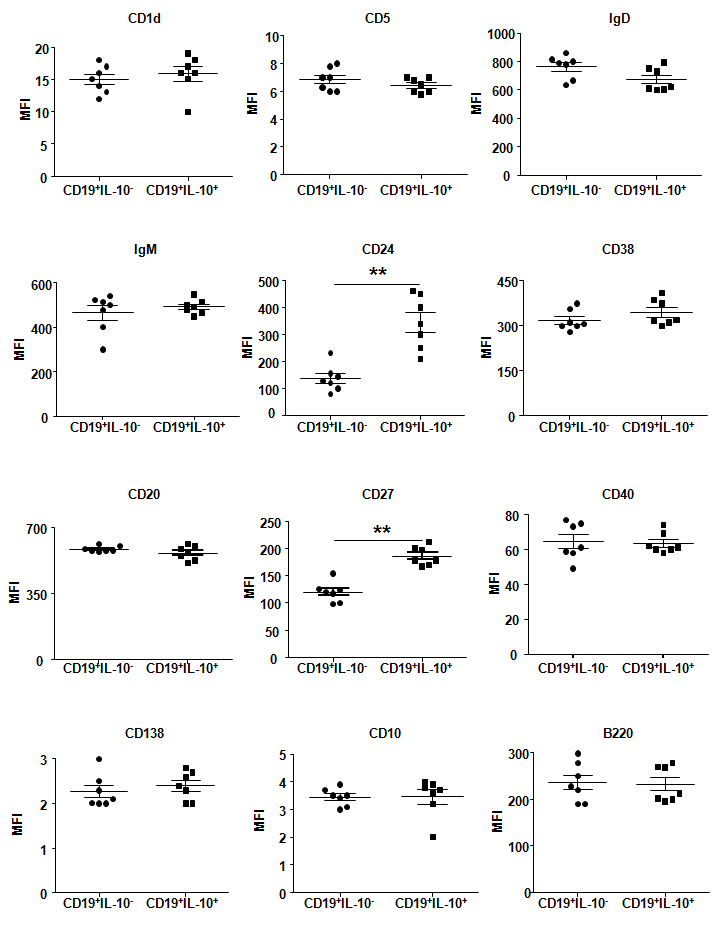


**Figure S1. Mean fluorescence intensities of the surface markers between IL-10+ and IL-10neg B cells.**

The mean intensity of ﬂuorescence (MIF) of CD1d, CD5, IgD, IgM, CD24, CD38, CD20, CD27, CD40, CD138, CD10 and B220 expression was analyzed by flow cytometry. Dots represent results of cell surface phenotypic analysis of IL-10^+^ (solid line) or IL-10^-^ (dashed line) B cells. Column and error bars represent mean±SEM. **p<0.001.
