# Supplementary material for: Decrease in Proportion of CD19+CD24hiCD27+ B Cells and Impairment of Their Suppressive Function in Graves’ Disease
Source: PLoS One. 2012 Nov 26;7(11):e49835. doi: 10.1371/journal.pone.0049835 (PMC3506658; doi:10.1371/journal.pone.0049835)
Supplement: Figure S2 — Frequencies of CD4+CD25hiCD127loFoxp3+ Tregs from the blood of healthy individuals and GD patients. (A) Representative intracellular Foxp3 expression in CD4+CD25hiCD127lo Tregs of healthy individuals, new-onset GD patients, and recovered GD patients. (B) Dots represent CD4+CD25hiCD127lo Tregs frequencies in CD4+ T cells and total PBMCs from 5 healthy individuals, 5 new-onset GD patients, and 5 recovered GD patients, respectively. Column and error bars represent mean±SEM. (DOC) [file pone.0049835.s002.doc]

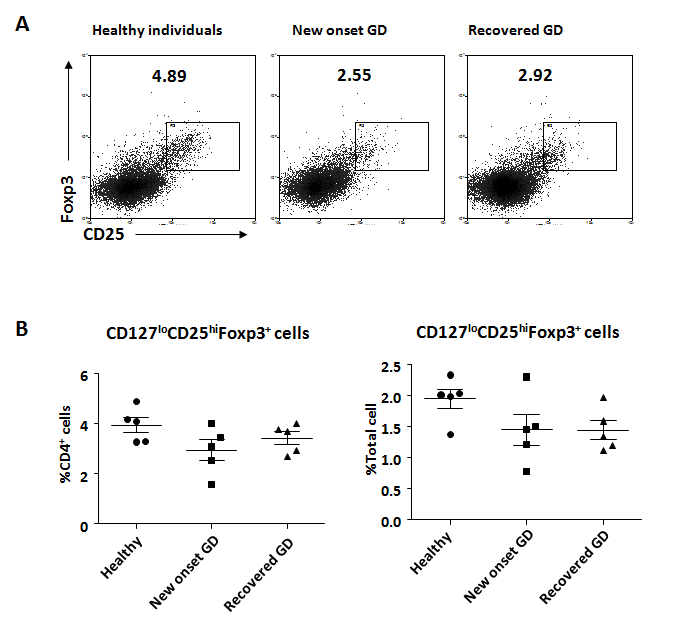


**Figure S2.** **Frequencies of CD4^+^CD25^hi^CD127^lo^Foxp3^+^ Tregs from the blood of healthy individuals and GD patients.**

(A)Representative intracellular Foxp3 expression in CD4^+^CD25^hi^CD127^lo^ Tregs of healthy individuals, new-onset GD patients, and recovered GD patients. (B) Dots represent CD4^+^CD25^hi^CD127^lo^ Tregs frequencies in CD4^+^ T cells and total PBMCs from 5 healthy individuals, 5 new-onset GD patients, and 5 recovered GD patients, respectively. Column and error bars represent mean±SEM.
